# Quantification of Surface Relation Between Experimental Polylactic Acid Dental Matrix and Type II Glass Ionomers Using Peel Adhesion Test and Fourier Transform Infrared Spectroscopy

**DOI:** 10.7759/cureus.35599

**Published:** 2023-02-28

**Authors:** Zeynep Ceren Çelik, Cigdem Elbek Cubukcu

**Affiliations:** 1 Restorative Dentistry, Bursa Uludag University, Bursa, TUR; 2 Pedodontics, Bursa Uludag University, Bursa, TUR

**Keywords:** three-dimensional (3d) printing, fourier-transform infrared spectroscopy, peel adhesion, dental matrix band, poly(lactic) acid

## Abstract

Aim: Type II glass ionomer cement (GIC) is a posterior restorative material that is generally not recommended for interaction with stainless steel due to chemical ion exchange. The purpose of this study is to quantify the surface relation of experimental three-dimensional (3D)-printed polylactic acid (PLA) and type II GIC using the peel adhesion test and Fourier transform infrared spectroscopy (FT-IR).

Materials and methods: Experimental PLA dental matrix specimens were 3D printed in the form of an open circumferential dental matrix (75x6x0.0055 mm) using a fused deposition modeling (FDM) machine. The peel resistance test (ASTM D1876) was applied to determine the relative peel resistance of the adhesive bonds between the PLA dental matrix, traditional circumferential stainless steel (SS) matrix, and GIC. The PLA bands were characterized using an FT-IR spectrophotometer (Spectrum 100, PerkinElmer Inc., Waltham, MA, USA) for the simultaneous determination of the chemical relationships of the surfaces before and after the GIC was set in a simulated class II cavity model.

Results: The mean peel strengths (P/b) ± standard deviations of the PLA and SS dental matrix bands were 0.0017 ± 0.0003 N/mm and 0.3122 ± 0.0042 N/mm, respectively. The -C H stretching was observed at 3383 cm^−1^ after adhesion, which corresponded to vibrational movements on the surface.

Conclusion: It required ~184 times less force to separate the GIC from the PLA surface compared to the traditional SS matrix. Additionally, there was no evidence of a new chemical bond or strong chemical interaction occurring between the GIC and the experimental PLA dental matrix.

## Introduction

Dental matrix bands are an essential tool in restorative dentistry and are commonly used to help dentists restore the interproximal surfaces of teeth. A matrix band is a thin strip of metal or plastic that is placed around the tooth to create a temporary wall that helps position restorative material properly. This process allows the dentist to properly shape and contour the restorative material to fit the anatomy of the tooth, creating a more natural appearance and improving the overall function of the tooth [1-7]. Matrix systems aid in the shaping of restorations and the maintenance of periodontal health, saving patients from potential subgingival irritation [2].

They also prevent adherence of the filling material to the adjacent tooth enamel and establish the optimum contact width between neighboring teeth [3,4]. Open contacts can result in vertical food impactions, which are one of the most common iatrogenic factors for the failure of posterior restorations [3,5-7].

Stainless steel (SS) is widely used in matrix systems in various thicknesses and types (circumferential, sectional, etc.) and has several advantages, such as being a contoured, reusable, and high-strength material [7,8]. While SS is a commonly used material as a dental matrix band, it can have some limitations when used as hand tools such as spatulas and mixing pads with chemically set glass ionomer or polycarboxylate cement.

Glass ionomer cement (GIC) is commonly referred to as a posterior restorative material to establish chemical bonds with hard dental tissues [9-11]. Sikka (&) Brizuela briefly explain the setting of GIC, which comprises four stages of ion exchange through an acid-base reaction, in a recently published book: dissolution (release of calcium (Ca2+), sodium (Na+), and fluoride (F-) ions), primary setting (three-dimensional (3D) cross-linked structure), final setting (formation of silica gel; aluminum (Al3+) ions and polyacrylic acid chains displace Ca2+ ions), and maturation (hydration of Al3+ and Ca2+ cross-linked polyacrylate chains) [12].

The GICs are hydrophilic, meaning they can wet the dental hard tissues and form long-lasting adhesive bonds via an acid-base reaction [11,13]. They are bioactive and have a wide range of uses, classified by type as follows [12]-type I: luting cement for prosthetic restorations; type II: direct, permanent restorative treatments; type III: cavity liners and bases; type IV: pit and fissure sealants; type V: luting cement for orthodontic appliances; type VI: core buildup in highly damaged teeth; type VII: fluoride-releasing, light-cured GIC; type VIII: atraumatic restorative treatment (ART); type IX: pediatric and geriatric restorations.

However, during the use of SS matrix bands at the proximal walls of the tooth to be restored using GIC material, the material undesirably adheres to them. Manufacturers recommend that metal spatulas not be used when preparing chemical powder-liquid GIC mixtures [11,12]. Because of the negatively charged chemical structure of SS matrices [14], temporary adhesion of GIC to the matrix band during the initial setting may result in the deterioration of the integrity of the dental filling (restoration) during the removal of the matrix band.

Polylactic acid (PLA), a 3D printable biopolymer, is a promising thermoplastic biomaterial for healthcare applications, including dental materials [15]. The PLA has hydrophobic surfaces and is chemically inert, with non-reactive side chain groups on its surface [16].

The present study aims to explore the surface relations of experimental PLA and traditional SS dental matrices and type II GICs using a peel adhesion test with a T-type specimen and Fourier transform infrared spectroscopy (FT-IR).

## Materials and methods

All procedures performed in this study were in accordance with the 1964 Helsinki Declaration and its later amendments or comparable ethical standards. The present in-vitro study was not considered within the scope of clinical and experimental studies on humans and animals by the Bursa Uludag University Faculty of Medicine Ethical Committee Board.

The PLA specimens (n=15) were 3D printed in the form of an open circumferential dental matrix (length: 75 mm; width: 6 mm; thickness: 0.055 mm) using a fused deposition modeling (FDM) machine, the Ender Pro 3 (Creality®, Shenzhen, China). The PLA (FK Filament, Istanbul, Turkey) specimens were printed with the nozzle temperature set at 200°C. The magnetic build plate temperature was kept constant at 60°C.

The ASTM D1876 adhesive peel resistance (T-peel test) was used to determine the relative peel resistance of the adhesive bonds between the dental matrices (PLA (n=15) and SS (n=15)) and the type II GIC.

The surfaces of the metal and experimental dental matrices were cleaned with an alcohol-free surface disinfectant and gently dried. The powder and liquid components of GIC (Ionofil U, VOCO GmbH, Cuxhaven, Germany) were mixed in a 4:1 ratio as the one scoop and one drop were prepared with a plastic spatula for 50 seconds. Homogenous cement was placed as a single coat between two layers of PLA and SS matrices. The thickness of the film (hA) was kept constant; thus, the test panels with different thicknesses were discarded. The variables of the standard test method for the peel adhesion (T-peel) test performed in this study are listed in Table 1. Thickness measurements were performed using a standard digital thickness gauge (SM-112, Teclock Co.Ltd., Nagano, Japan).

**Table 1:** Variables of the standard test method for peel adhesion (T-peel) test PLA: Polylactic acid, SS: Stainless steel, SD: Standard deviation, hA: Thickness of the film

	PLA (n=15)	SS (n=15)
Peel angle (^0^)	180	180
Test speed (mm/min)	10	10
Specimen dimensions (length/width; mm/mm)	75/6	75/6
Gauge length (mm)	51.2	51.5
Thickness of adhesive layer hA (mean ± SD (µm))	244.2 ± 33.1	256 ± 44.4

**Figure 1:**
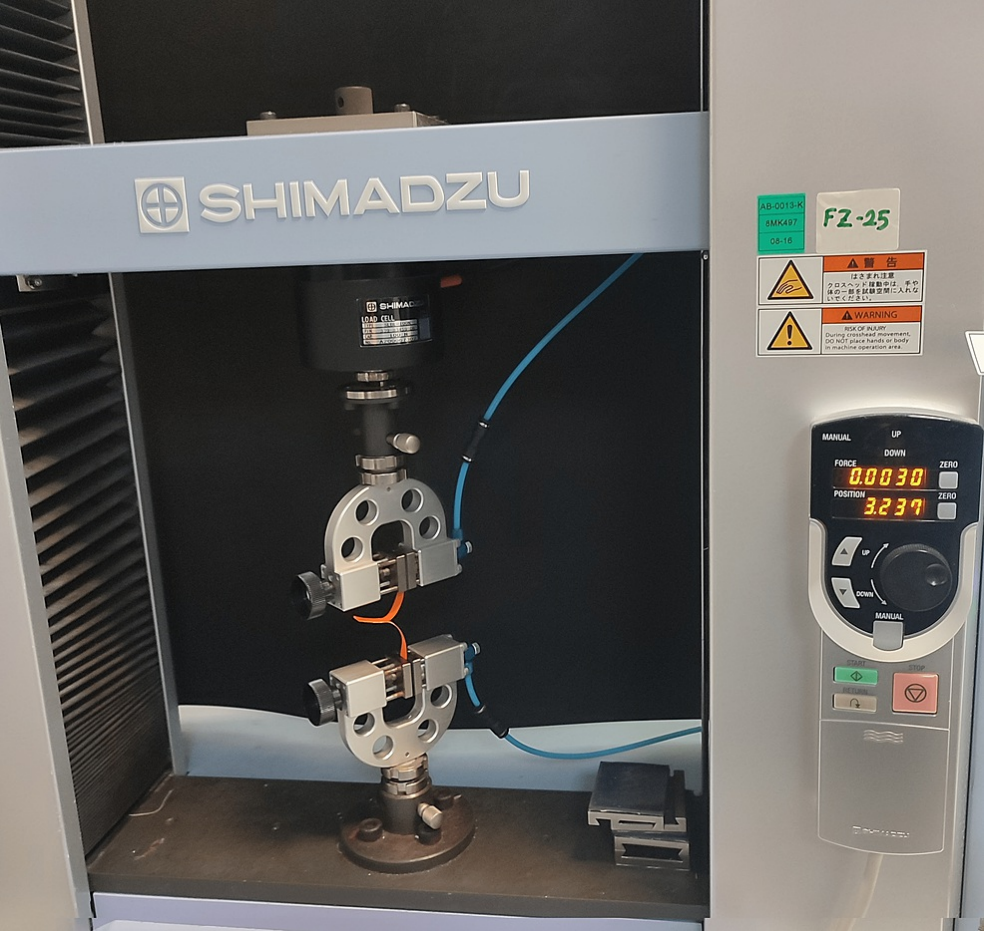
The experimental setup for the peel adhesion test (T-type specimen)

Each end of the T-peel specimen was clamped in separate test grips on a universal tester machine (EZ-SX, Shimadzu, Kyoto, Japan). The stretching speed was 10 mm/min, and the test was conducted at 22.5 ± 1°C with a relative humidity of 50 ± 2% (Figure 1).

All trials were repeated 10 times, and mean values were recorded. The PLA bands were characterized using an FT-IR spectrophotometer (Spectrum 100, PerkinElmer Inc., Waltham, MA, USA) before and after the GIC was set in a simulated class II cavity model. The measurements were performed using the attenuated total reflectance (ATR) technique. Each sample was scanned 32 times at a resolution of 2 cm-1 over a frequency range of 4000-500 cm-1.

## Results

**Figure 2:**
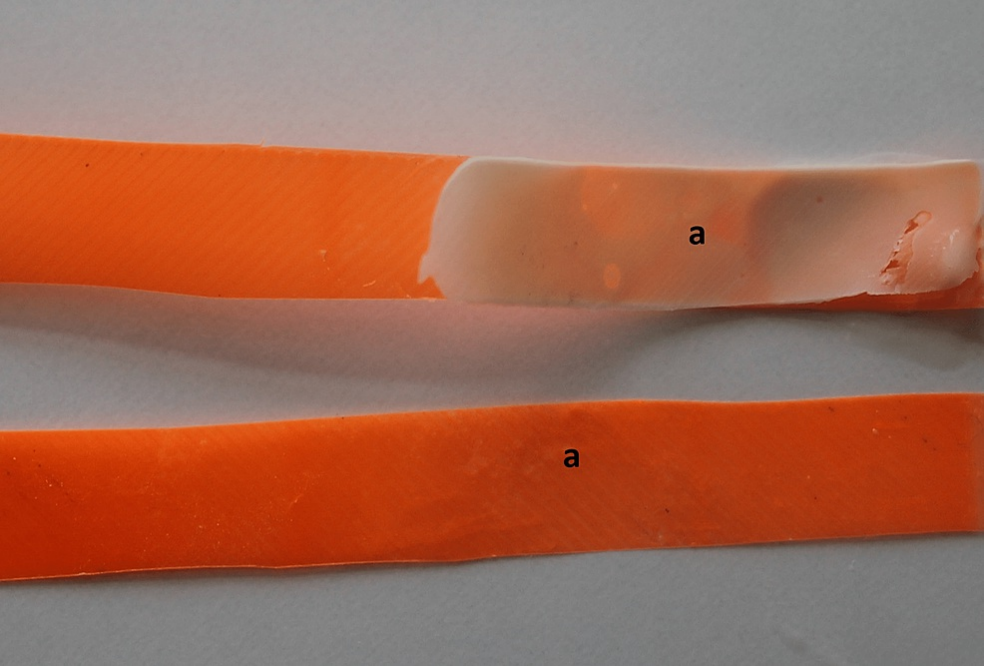
Adhesive failure of PLA dental matrix after peel adhesion test a: adhesive failure separation PLA: Polylactic acid

**Figure 3:**
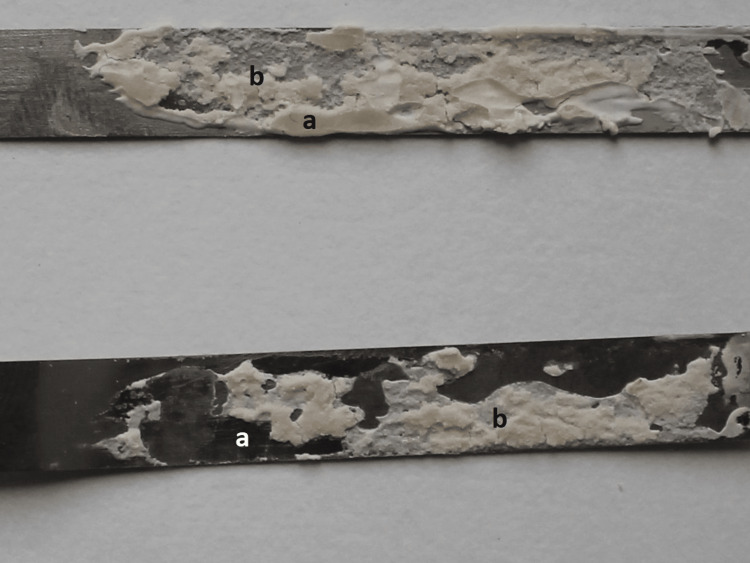
Adhesive (a) and cohesive (b) failure of SS dental matrix after peel adhesion test SS: Stainless steel

The mean peel strengths (P/b) ± standard deviations of the PLA and SS dental matrix bands were 0.0017 ± 0.0003 N/mm and 0.3122 ± 0.0042 N/mm, respectively. Evaluating the type of failure pointed to only adhesive failure that was observed in a PLA dental matrix (Figure 2) cohesive and adhesive debonding of GIC in a SS matrix (Figure 3). 

Comparing the initial volumetric thickness of GIC with the remnants on the separated plates of SS, 45% of an adhesive material remained bonded on both internal surfaces of the SS matrix bands. However, there were no GIC remnants on the surface of PLA dental matrices after debonding.

**Figure 4:**
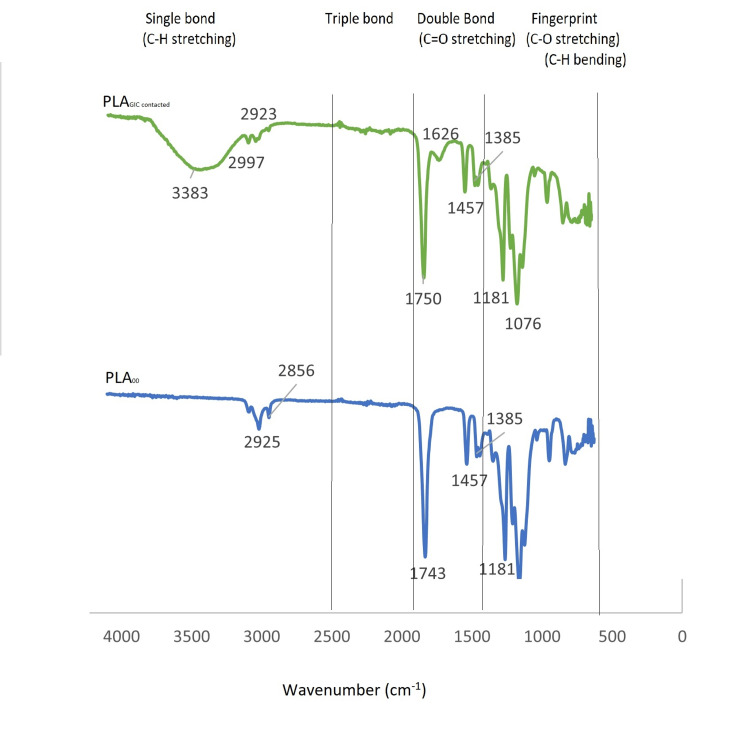
FT-IR spectra of PLA dental matrices before (PLAoo) and after GIC (PLA-GIC contact) application FT-IR: Fourier transform infrared spectroscopy, PLA: Polylactic acid, GIC: Glass ionomer cement

Figure 4 shows the FT-IR spectra of the PLA dental matrices before and after the GIC application. The PLA dental matrix’s characteristic frequencies were 2997, 1750, and 1076 cm^-1,^ which correspond to C-H stretching, C=O stretching, and C-O stretching, respectively. The C-H bending frequencies are detected between 1500 and 500 cm^−1.^ A broad peak was observed at 3383 cm^−1 ^after adhesion, which corresponded to vibrational movements on the surface. Therefore, no new bonds were formed or strong chemical interactions occurred after GIC was applied to the PLA matrix.

## Discussion

The use of a polymer-based 3D printed matrix has been shown to provide a lower bonding tendency than type II GIC. During the setting process of GIC, an ion-exchange interface is formed with the dental tissue by the reaction of the glass particles (base) and polyacrylic acid. The bonding tendency of GICs used in restorative treatments to the tooth is highly desirable. Besides, this feature makes GIC a commonly preferred material as a luting agent for orthodontic brackets and pediatric SS crowns [17,18]. However, ion interactions occur between positively charged ions released from GIC and negatively charged stainless steel [14,19]. Thus the attachment to dental instruments and tools often made of SS can be challenging in dental practice [11]. Restorative materials temporarily adhere to SS bands used in the restorations of proximal cavities; thus, during band removal, the newly made restoration may lose its integrity in the interproximal area. The search for a new product, which was the focus of the present study, guided the selection of PLA which is reported to be chemically inert and uncharged in the literature [16,20]. The PLA also has several advantages over SS, such as being easy to manufacture, 3D printable, biodegradable, and biocompatible [15,21].

To the best of our knowledge, there is no commercially available dental matrix band made of PLA, and there is no literature on its adhesive behavior towards glass ionomers. The peel test can be used successfully to assess interface adhesion and can be used as a fast, easy, and reliable test to study the long-term durability of bonded surfaces. It evaluates the maximum force required to separate two bonded surfaces, which correspond to 3D-printed PLA plates and SS circumferential matrices in our study.

In a recent review, it was stated that the peel angle might influence peel resistance; however, there was no correlation between bonding tendency and adhesive layer thickness [22]. In the present study, the conditions and variables were kept the same for the experimental and control groups to prevent any bias. In our study, it was determined that the PLA plates were separated from each other by a force of approximately 184 times less than that of traditional SS. This indicated that PLA might be a suitable dental matrix for proximal glass ionomer restorations.

In addition, changes in the surface organic layer of PLA upon contact with GIC at the molecular level were analyzed using FT-IR spectroscopy. Infrared spectroscopy is a powerful technique for identifying the functional groups present in a molecule. The carbonyl stretching vibration is one of the most important infrared absorptions, and it typically appears in the range of 1630 to 1780 cm^-1^ [23]. In this study, a small peak corresponding to C=O stretching was observed on the surface of PLA after contact with GIC at 1626 cm^-1^. 

Stretches and bends correspond to increasing and decreasing bond lengths within a molecule and angles between bonds in a molecule, respectively. In our study, the only significant difference between the initial PLA and contacted PLA was detected between 3500 and 3000 cm^-1^ by infrared spectroscopy, which corresponds to C-H stretching.

The C-H stretching is considered a vibrational movement that does not cause a profound chemical change in the surface [24]. This result indicates that the PLA surfaces did not contain any GIC remnants after usage, verifying their non-adhesive features. In a present study, the broad peak at 3383 cm-1 might correspond to vibrational C-H stretching.

Functional applications on PLA during 3D printing change the chemical nature by increasing its mechanical and physical features and transforming it more susceptible to water, alcohol, acetone, and heat [25]. The major limitation of the study might be the challenge of disinfection of 3D-printed PLA without any chemical alteration and degradation. However, Cuiffo et al. suggested future implications on ethylene oxide sterilization for 3D-printed PLA [25]. 

A present study offers a new personalized/customized dentistry application with predictable and repeatable prints designed by software. In addition, existing design and printing settings can be shared with other researchers, and further multi-center studies can be carried out simultaneously.

## Conclusions

This study is a pioneer in restorative dentistry as an exploration of novel biocompatible and biodegradable 3D-printed dental matrix bands. The peel adhesion test quantitatively revealed the adhesive strengths between GIC and dental matrix groups with a low standard deviation. Given that both cohesive and adhesive failures were detected in SS-GIC-SS peel adhesion, highly operative caution is needed during the removal of traditional SS matrices after the initial setting. In addition, FT-IR outcomes presented no profound chemical changes on the surface of the GIC-contacted PLA dental matrix. The PLA dental matrix is not commercially available and might be a promising material for manufacturing showing a low adherence tendency for type II GIC under the conditions of the present in-vitro study. Clinical implications are needed in further studies.
